# Individual and community-level factors influencing optimal breastfeeding: A multilevel analysis from a national survey study of Ethiopia

**DOI:** 10.1371/journal.pone.0241428

**Published:** 2021-04-29

**Authors:** Amanuel Hagos, Mache Tsadik, Abate Bekele Belachew, Afewerki Tesfahunegn

**Affiliations:** 1 Tigray Regional Health Bureau, Adi-Daero Health Office, Adi-Daero, Ethiopia; 2 School of Public Health, College of Health Sciences, Mekelle University, Mekelle, Ethiopia; 3 School of Public Health, College of Medicine and Health Sciences, Arba Minch University, Arba Minch, Ethiopia; University of Cape Coast, GHANA

## Abstract

**Background:**

Optimal breastfeeding is critical for healthy growth of the child. Globally, 820,000 children and 20,000 women lost due to in appropriate breastfeeding each year. In Ethiopia, 50,000 children lost related to malnutrition with 18% were due to poor breastfeeding habit. Little is known on the determinants of breastfeeding with hierarchical level. Therefore, this study aimed to identify factors influencing optimal breastfeeding among children under six month in Ethiopia using a multilevel analysis.

**Methods:**

The data of this study were obtained from Ethiopian Demographic and health survey conducted from January to June 2016. A total 1,087 children aged 0–5 months were selected using two stage stratified sampling technique. Multilevel logistic regression analysis was done to identify significant explanatory variables. Akaike information criteria were used to select the best model fit. Fixed effect was done to estimate the association between the outcome and explanatory variable and also random effect to measure the variation explained by the higher level.

**Result:**

Among the total of 1,087 children, 45.4% were optimally breastfeed. Children from the richest wealth index (AOR = 2.87; 95% CI: 1.53–5.43) was positively associated with optimal breastfeeding but, children aged 4–5 months (AOR = 0.19; 95%CI: 0.12–0.27), children born through cesarean section (AOR = 0.18; 955 CI: 0.07–0.51) and residing in Afar region (AOR = 0.13; 95%CI: 0.02–0.92) were found inversely associated with optimal breastfeeding. The random-effects showed that the variation between communities was statistically significant.

**Conclusion:**

Individual and community level factors play a significant role in shaping optimal breastfeeding. Future strategies and health interventions should be strengthen to target individual and community level factors that enhance optimal breastfeeding.

## Introduction

Optimal Breast Feeding (OBF) is breastfeeding within one hour of birth (early initiation of breastfeeding (EIBF)), breastfed exclusively for the first six months of life, and continues to be breastfed up to two years of age [1]. OBF protects the two leading causes of child death pneumonia and diarrhea. Other than this, OBF builds a child's immune system that helps to prevent overweight/obesity and enhances mental development and bonding between mother and child [2–4]. The united nation on sustainable development goal planned to end hunger by 2030 providing nutritious and sufficient food including early initiation and exclusive breastfeeding for preventing malnutrition and death [5].

Breast milk makes the world healthier and exclusive breastfeeding can save 520,000 children’s live over the next 10 years [3–6]. Breastfeeding is cost-effective to ensure the well-being of the citizen and plays a role in poverty reduction [1, 7] However, about 68 countries have breastfeeding rates of below 50% in 2016 globally. Moreover, about 20% of child deaths each year are attributable to poor breastfeeding habits [4, 8].

In Sub Saharan Africa breastfeeding is estimated to be 41% and with the overall prevalence of initiation of breastfeeding within one hour and exclusive breastfeeding of 52.83% to 49.17% respectively [9]. In Ethiopia, the prevalence of exclusive breastfeeding is 52% and about one-third of babies do not receive breastfeeding within one hour of birth and only one-third of children 4–6 months old received exclusively breastfeeding [4]. One study in south Ethiopia also reveals that the prevalence of OBF was found that 37.3% [10].

Optimal breastfeeding can be affected by giving the child an early additional food or fluid which is about 28.92% in Ethiopia. There are 50,000 child deaths a year related to malnutrition. Of these, about 9,000 (18%) deaths are linked to poor breastfeeding habits. Moreover, about 38% of children under age five are stunted, 10% are wasted, and 24% are underweight [11]. If optimal breastfeeding wasn’t achieved, the child can suffer from irreversible growth and die from diseases like diarrhea and pneumonia [4].

Different literatures in Ethiopia, Africa, and globally; identified that individuals factors like children’s age [12, 13], mode of delivery [13, 14], weight of children [15–17], maternal status age [13], marital status [17], education [12–14], religion [9, 16], ethnicity [9, 16], antenatal visits [14, 18, 19], place of delivery [13, 16], wealth index [19], and media exposure [19] were the associated factors of OBF. Also, factors like residence [13, 15], region [15, 16], community ANC coverage [9], and community media exposure were some of the community factors associated with OBF [19, 20].

The previous studies in Ethiopia mainly concentrated on individual-level factors and analysis was made using ordinary logistic regression. However, as EDHS data has a hierarchical nature, a multilevel logistic regression analysis is preferred to consider the individual and community level factors simultaneously and there are limited studies based on this multilevel analysis. The result will help policy makers, program officer and local authorities to map the community with optimal breastfeeding and to mitigate malnutrition and end hunger by 2030. Thus, this study aimed to identify factors associated with optimal breastfeeding among children age under six month in Ethiopia.

## Materials and methods

### Study area, design and population

**Fig 1:**
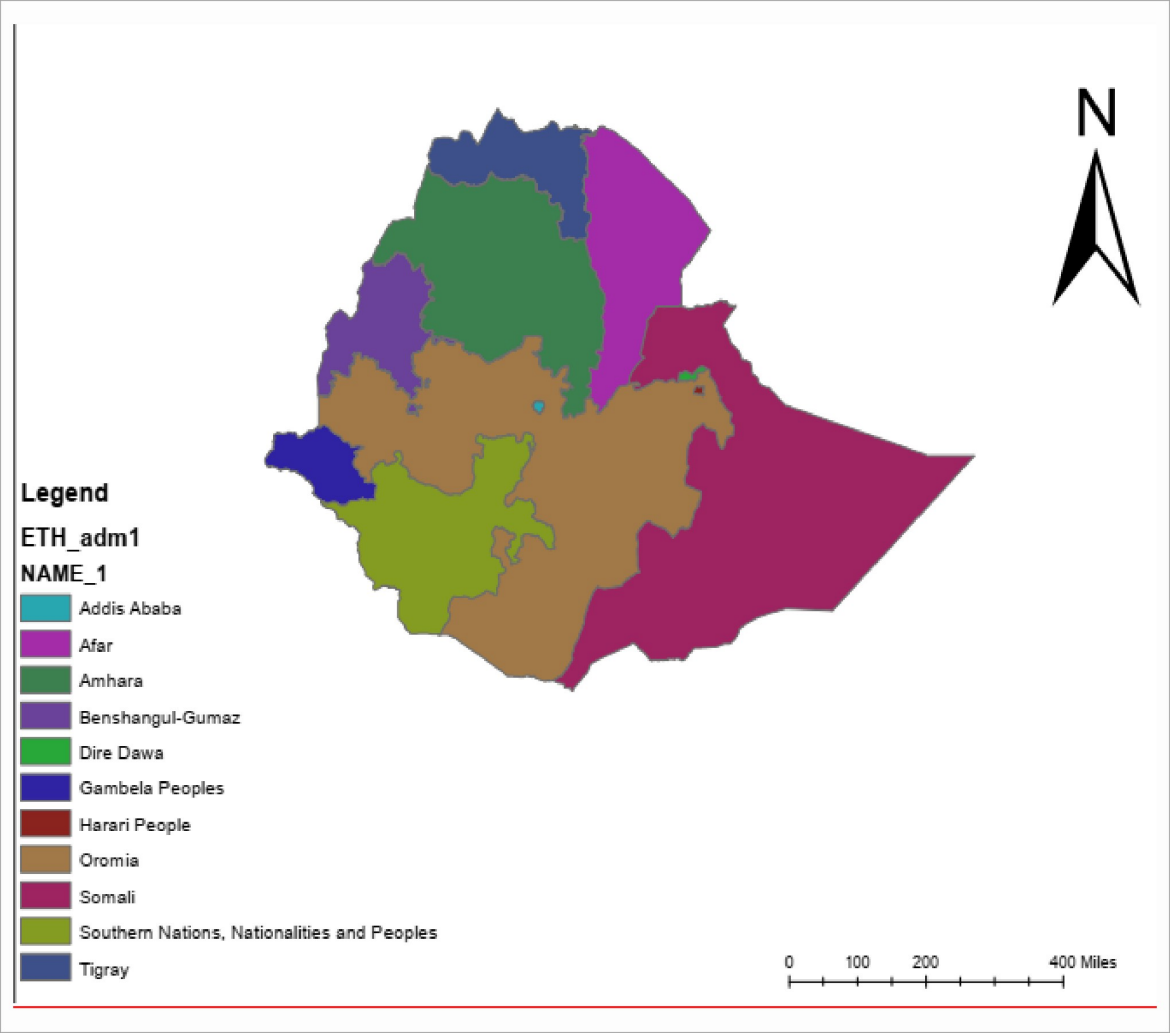
Map of Ethiopia with regions, 2019.

The study was conducted in Ethiopia located in the horn of Africa with a total population of about 105,350,020 [21]. The country has 9 Regional States and two city administrations with over 80 different ethnic groups (Fig 1). About 65% of rural households in Ethiopia consume the World Health Organization's minimum standard of food per day (2,200 kilocalories), with 42% of children under 5 years old being underweight [22]. This study employed a cross-sectional study design to assess the determinants of OBF among a total of 1,087children aged 0–5 months in EDHS 2016 selected enumeration areas [23]. All women of the reproductive age group residing in the selected households were eligible to be interviewed. All the data related to all children age 0–5 months in the EDHS 2016 were included in this study.

### Sample size determination and procedure

Each region was stratified into urban and rural areas yielding 21 sampling strata. Samples of enumeration areas were selected independently in each stratum in two stages. In the first stage, a total of 645 enumeration areas (EAs) (202 in urban areas and 443 in rural areas) were selected. In the second stage, a fixed number of 28 households per EAs were selected systematically from newly created household list by equal probability. All mothers/care givers of 0–5 month infants were eligible for the interview. A total of 1,087 children’s from selected house hold were included in the study.

### Data source, data extraction, and management

The data source was EDHS, 2016 which is nationally representative large scale dataset conducted by the central statistical agency (CSA) in collaboration with the ministry of health from January 18 2016, to June 27, 2016 from all nine regions and two administrative cities [23]. The data set was obtained by attaching the title and significance of the study. Then, after receiving permission for registration data set was downloaded at www.measuredhs.com from Measure DHS website. This was followed by the extraction of a wide range of information about potential individual and community level factors from the EDHS 2016; particularly from the dataset of child records including child mortality, nutrition, maternal, child health, family planning and other reproductive health issues.

For the 2016 EDHS, prior to that at the start of data collection training was given for staff and supervisors and a manual that describe the mapping procedures was prepared. Rounds of supervision were carried out by CSA central office staff to assess the quality of the field operation. In addition, standard data collection tool was used and the questioner was pre tested [10–14]. After downloading from Measure DHS data cleaning, labeling, coding was done for all selected variables. Categorization was done for continuous variables using information from different literatures.

### Variables and measurements

Optimal breastfeeding: refers to a child less than six months old who had received breastfeed timely (within one hour of birth) and had no intake of food/fluids other than breast milk for the first six months.

In this study, the dependent variable was optimal breastfeeding (OBF) practices. In the regression analysis, OBF practice was coded ‘1’ while ‘0’ was coded for non-OBF practices. The explanatory variables considered were age, residence, educational status, marital status of mothers, household income, occupation, family size, sex of the infant, place of delivery and ante-natal and post-natal service utilization [S1 Appendix].

### Data analysis

The extracted dataset was cleaned, coded, and analyzed by using Stata version 13.1 and excel was used. After generating the new variables EIBF and EBF the outcome variable was also generated from both EIBF and EBF. EDHS sample was not self-weighted because of non-proportional allocation. Therefore, sampling weights were used to make the sample representative of the entire population.

Frequency and percentage were used to report categorical variables, while median was used to report non-parametric continuous explanatory variables. Bivariate analysis was performed to examine the association between optimal breastfeeding and each individual (level 1) and community-level factors (level 2) at p-value less than or equal to 0.25. Finally, multivariable multilevel logistic regression analysis was performed to estimate the adjusted odds ratios for both levels and to estimate the extent of random variation between communities at a p-value of less than 0.05 and confidence interval 95% [24].

Assuming varying intercept across communities but fixed coefficients, four models were developed. The first one is the Null model; this is a model with no explanatory variables whereas Model I is a model with only individual level variables. Model II is a model constructed with community level variables only and Model III is a combined model which is developed by combining both the individual and community level variables together. Null model (the model with no explanatory variables) was fitted to estimate the clustering effect or between community variation and to justify the application of multilevel analysis by determining ICC. Accordingly, about 20% of the total variation in the odds of optimal breastfeeding children is due to community difference. This high ICC value and significant community variance (P*<*0.05) justifies that the application of multilevel model is appropriate (Table 1).

**Table 1:** Random effects and model fitness of individual and community-level factors associated with optimal breastfeeding among 0-5-month-old children, Ethiopia 2016.

Random effect	Null Model	Model I	Model II	Model III
Community variance (SE)	0.797* (0.274)	0.615* (0.271)	0.399* (0.219)	0.389* (0.252)
ICC (%)	19.5	15.75	10.82	10.6
PCV (%)	Reference	22.8	49.9	51
**Model fitness**				
Log likelihood	-718.445	-629.651	-689.84904	-592.05634
AIC	1440.892	1309.315	1407.698	1280.113

ICC: Intra-class correlation coefficient

*significant at p < 0.05

PCV: Proportional Change in Community Variance

AIC (Akaike information criterion)

The random effect in the final model showed that 51% of the variation in optimal breastfeeding across communities was explained by both individual and community-level factors. Accordingly; the model with the highest PCV (51%) expresses the change in the community level variance very well. The subsequent decrease in the values of AIC on each model indicates that the final model AIC (1280.113) was the best explanatory model fitting the data very well (Table 1).

The effect of multicollinearity between the predictor variables was checked using a variance inflation factor (VIF) at a cutoff point of 10. Predictors having a VIF value of less than 10 indicate an absence of Multicollinearity (S2 Appendix). Accordingly, no variables had VIF more than 10, indicating absence of significant collinearity.

### Ethical clearance

Ethical approval was obtained from Mekelle University College of Health Sciences (ERC = 1475/2018) and permission was also obtained for CSA to access the 2016 EDHS dataset was obtained from (web address: **http://www.measuredhs.com**), after making a request via DHS program website from CSA (S1 File).

## Results

### Description of individual and community level characteristics

Among the total of 1,087 surveyed children, 45.4% were optimally breastfed. About 35% of the children were in the age group of 4–5 months and 51% were female children. About 60.6% children below the age of less than 2 months were optimally breastfed. Optimal breastfeeding was very low among mothers with the educational status of secondary school and above (Table 2).

**Table 2:** Distribution of OBF with individual level variables among 0–5 month old children, Ethiopia 2016 (n = 1,087).

variables	Category	Frequency	OBF (%)
Sex of child	Male	532	227(42.7)
	Female	555	266 (47.9)
Age categories of children	<2 month	353	214 (60.6)
	2–3 month	352	187 (53)
	4–5 month	382	93 (24)
Size of a child at birth ^a^	Large	261	116 (44.4)
	Average	467	220 (47)
	Small	359	157 (43.7)
Birth order	1^st^	242	87 (35.9)
	2–4	457	231 (50.5)
	Five and above	388	175 (45)
Mothers educational status	No education	642	286 (44.5)
	Primary education	418	200 (47.8)
	Secondary and higher	27	7 (24)
Mothers occupation	Not employed	868	395 (45.5)
	Employed	219	98 (44.7)
Marital status	Never married	9	1 (11)
	Married/living together	1,051	481 (45.7)
	Divorced/widowed/separated	26	11 (42.8)
Wealth index	Poorest	268	102 (37.9)
	Poor	255	135 (53)
	Middle	195	97 (49.7)
	Richer	201	70 (34.8)
	Richest	168	89 (53)
Parity of the mother	1–2	411	179 (43)
	3–4	288	138 (48)
	Five and above	388	176 (45.4)
ANC utilization	No ANC visit	371	174 (46.7)
	1 visit	61	19 (31.8)
	2–3 visit	310	151 (48.7)
	> = 4 visits	336	149 (44)
Place of delivery	Home	672	308 (45.8)
	Health facility	397	177 (44.6)
	Other	18	8 (44)
Mode of delivery	Normal	1,055	487 (46)
	Cesarean section	32	6 (17)
Husband education	No education	505	228 (45)
	Primary education	384	187 (48.5)
	Secondary and higher	162	66 (40.7)
Husband occupation	Not employed	74	27 (36.3)
	Employed	978	454 (46.4)
Ethnicity of mother	Amhara	235	113 (48)
	Oromia	478	217(45.3)
	Tigray	76	36 (47.3)
	Afar	8	1 (13)
	Somalia	48	10 (20.8)
	Others	242	114 (47)

^a^ Based-on mother’s estimate of baby’s size at birth, ANC: Antenatal care

Relatively, mothers from Afar and Somali regions reported a low proportion of optimal breastfeeding. There is no significant variation in the proportion of optimal breastfeeding in the other community-level variables (Table 3).

**Table 3:** Distribution of OBF with community level variables among 0–5 month old children, Ethiopia 2016 (n = 1,087).

variables	Category	Frequency	OBF (%)
Community media exposure	Low	482	233 (48.3)
	High	605	260 (43)
Community education	Low	560	250 (44.6)
	High	527	243 (46.1)
Region	Tigray	76	36 (47.5)
	Afar	9	2 (22.2)
	Amhara	204	96 (47)
	Oromo	495	224 (45.2)
	Somalia	50	13 (23.6)
	Benshangul	12	6 (50)
	SNNP	207	102 (49.2)
	Gambela	2	1 (50)
	Harare	3	1 (33.3)
	AddisAbeba	25	10 (40)
	Dire Dawa	4	2 (50)
Place of residence	Urban	124	57 (46)
	Rural	963	436 (45)
Community ANC	Low	514	242 (47)
	High	573	251 (43.9)
Community poverty	Low	623	276 (44.3)
	High	464	217 (46.7)
Community place of delivery	Low	532	233 (43.8)
	High	555	260 (46.8)

ANC = Antenatal care

### Multilevel factors influencing optimal breastfeeding

In the final model, age of children, wealth index, delivery by c/section and region were found significantly associated with optimal breastfeeding. After controlling the potential confounders at the individual and community level factors, the odds of optimally breastfeeding were higher among children from the richest family (AOR = 2.87; 95% CI: 1.53–5.43) compared to those from the poorest family. Children 4–5 months old had lower odd of optimal breastfeeding compared to those whose age of less than two months (AOR = 0.19; 95% CI: 0.12–0.27). Children born with a cesarean section had 82% lower odds (AOR = 0.18; 95% CI: 0.07–0.51) of breastfeeding optimally than their counterparts born other than cesarean section. Children born from mothers residing in the Afar region had 87% lower odds (AOR = 0.13; 95% CI: (0.02–0.92) to breastfeed optimally than children born in Addis Ababa (Table 4). The mean VIF value of 3.07 indicated the absence of collinearity between explanatory variables.

**Table 4:** Individual and community-level factors associated with OBF among 0-5-month-old children, Ethiopia 2016 (n = 1,087).

Variables	Category	Model I	Model II	Model III
		AOR [95%CI]	AOR [95%CI]	AOR [95%CI]
Child age group	<2 month	1.00		
	2–3 month	0.86(0.60–1.22)		0.85(0.59–1.21)
	4–5 month	0.19 (0.13–0.28)*		0.19(0.12–0.27)*
Wealth index	Poorest	1.00		
	Poorer	1.52 (0.95–2.43)		1.38(0.85–2.26)
	Middle	1.68 (0.99–2.86)		1.56(0.90–2.71)
	Richer	0.91 (0.54–1.54)		0.91(0.52–1.59)
	Richest	2.16 (1.27–3.70)*		2.87(1.53–5.43)*
Mode of delivery	None C/S	1.00		
	C/ S	0.19 (0.07–0.50)*		0.18(0.07–0.51)*
Ethnicity of mother	Afar	1.00		
	Amhara	7.39 (2.87–19)*		
	Oromia	4.93(2.17–11.19)*		
	Tigray	6.89(2.48–19)*		
	Somalie	2.14 (0.92–5.05)		
	Others	5.61(2.37–13.44)*		
Region	Addis Ababa		1.00	
	Tigray		1.31(0.60–2.83)	0.87 (0.13–5.81)
	Afar		0.29(0.11–0.73)*	0.13 (0.02–0.92)*
	Amhara		1.57(0.71–3.50)	1.32(0.44–3.91)
	Oromo		1.56(0.72–3.41)	1.87(0.66–5.34)
	Somalie		0.60 (0.27–1.35)	1.94(0.37–10.3)
	Benshangul		1.74(0.76–3.98)	1.69 (0.61–4.71)
	SNNP		1.75(0.80–3.82)	2.12 (0.72–6.27)
	Gambela		0.97(0.39, 2.38)	1.50 (0.44–5.13)
	Harare		0.85(0.34–2.11)	0.74(0.24–2.29)
	Dire Dawa		1.87(0.75–4.68)	2.63(0.84–8.30)

*significant at p < 0.05 C/s = caesarian section

## Discussion

The study aimed at identifying individual and community level factors that influence optimal breastfeeding among children 0-5months. Variables such as the age of child, wealth index, delivery by c/section and region were found statistically significant predictors of optimal breastfeeding.

The current study showed that, the magnitude of optimal breast feeding was 45.4%. This result is lower comparing to WHO and UNICEF guidelines [1, 2, 4, 5, 9]. This could be due to the fact that our study measures both early initiation of breastfeeding and exclusive breastfeeding. And higher comparing to the study conducted in Amhara Region of Ethiopia [10]. This could be due to the difference in study area and population group. This study was conducted among both urban and rural community.

As shown in this study, the age of children was inversely associated with optimal breastfeeding. Children’s 4–5 months were less likely to breastfeed optimally when compared to the age of less than two months. Possibly, as the age of the child increases the practice of optimal breastfeeding decreases which is consistent with the studies in Ethiopia [10, 19]. Similarly, studies in other countries like Tanzania, Zimbabwe and Nepal showed that the practice of optimal breastfeeding likely reduces with the increasing age of children [12–13, 25]. This might be related to the misunderstanding of mothers about the importance of optimal breastfeeding and the initiation of additional food when the child’s age increases. Likewise, another possible explanation for less optimal breastfeeding may be related to the perception and cultural practice of mothers that breast milk production is insufficient for the child’s growth [25, 26].

In the current study, delivery by cesarean section has less likely to be optimal breastfeeding compared to children delivered without cesarean section. This finding is consistent with previous reports from Ethiopia and Tanzania [10, 13]. The possible explanation can be related to the effects of anesthesia delaying the onset of lactation and some baby-unfriendly postoperative-care practices. During cesarean delivery, more attention may be given to the mother, while the child feeding might be forgotten. Mothers might also be uncomfortable to breastfeed because of the pain experienced after surgery or take longer to recover from the effects of the anesthesia [16, 17]. This might cause a delay in making the first contact with the child, and mothers might also find it difficult to achieve comfortable breastfeeding positions. In addition, children born by cesarean section might have respiratory distress. Hence, they are more likely to be taken to a newborn intensive care unit and physically separated from their mother [15, 18].

In this study, the wealth index has a positive relationship with optimal breastfeeding. Children from the richest wealth index were almost 3 times more likely to be optimally breastfeed compared to children from the poorest wealth index. This finding is similar to the study conducted by the Centers for Disease Control and Prevention and National Center for Health Statistics which indicates the poor practice of optimal breastfeeding among mothers in the poorest wealth index [27] In contrast to this, other studies have shown that mothers with the poorest wealth index practice optimal breastfeeding compared to the richest wealth index [10, 19, 20, 28]. The low practice of optimal breastfeeding among the poorest could be linked with the lack of awareness, stressful living situations to overcome the hard of living [27]. Likewise, it can also be explained as mothers from the poorest index might consider themselves as producing inadequate breast milk that satisfies their child’s demand which influences them to look for additional food.

Among the community level variables, the region was found significantly associated with optimal breastfeeding. Children from Afar were less likely to optimally breastfeed than children from Addis Ababa. This is contextually similar to other studies conducted in Ethiopia, Nigeria and Nepal [15, 16, 29]. Mothers residing in towns and villages practice less optimal breastfeeding compared to those residing in larger towns and cities like Addis Ababa. The reason behind is those residing in larger towns and cities may have better access to health information and healthcare service. Besides, mothers residing in larger towns and cities may be less influenced by traditional and cultural practices than their counterparties.

### Strength and limitation

As this study was nationally representative data, the study findings can be used to inform policy and program actions. In addition, this study applied multilevel modeling to accommodate the hierarchical nature of the EDHS data and to examine the extent of variations across communities. Despite the strengths, the data used for this analysis were from a cross-sectional survey. As a result, only associations were examined and may not draw a true conclusion about causality and may increase also recall-bias. Also, some important variables such as maternal beliefs, miss-conceptions and knowledge towards breastfeeding were not included.

## Conclusion

The magnitude of OBF was low in this study. Regarding the predictors of OBF, both the individual and community level factors were found to be significantly associated. At the individual level; children’s age, wealth index and mode of delivery were the factors that influence optimal breastfeeding. Among the community characteristics, only region was found as a significant predictor of OBF. Thus, interventions to create awareness about the benefits of OBF should be strengthening from the previously implemented activities in areas where there is low practice of OBF. Moreover, Mothers delivered by CS should be assisted and counseled to breastfeed their child within the recommended time by the care providers.

## Supporting information

S1 File Authorization letter. (PDF)

S2 File Child dataset final. (DTA)

S3 File STROBE check list. (DOCX)

S1 Appendix (DOCX)

S2 Appendix (DOCX)
