# Supplementary material for: Individual and community-level factors influencing optimal breastfeeding: A multilevel analysis from a national survey study of Ethiopia
Source: PLoS One. 2021 Apr 29;16(4):e0241428. doi: 10.1371/journal.pone.0241428 (PMC8084135; doi:10.1371/journal.pone.0241428)
Supplement: S3 File — (DOCX) [file pone.0241428.s003.docx]

Check list based on STROBE statement of cross sectional study

| **Activities** | **Recommendation for referral** | **Remark** |
| --- | --- | --- |
| **Title and abstract** | “Title page, page 1, line 1-2” |  |
|  | “abstract, page 2, line 20-43” |  |
| **Introduction** | | |
| Background/rationale | “Introduction, page 3-4, line 44-76/ page4, line 77-82” |  |
| Objectives | “Introduction, page 4, line 82-83” |  |
| **Methods** | | |
| Study design | “Method, page 5, line 91-92” |  |
| Setting | “Method, page 5, line 87-88” |  |
| Participants | “Method, page 5, line 92-96” |  |
|  | (*b*)Not applicable |  |
| Variables | “Method, page 6/7, line 122-130” |  |
| Data sources/ measurement | “Method, page 6, line 105-121” |  |
| Bias | “Method, page 6, line 105-121” |  |
| Study size | “Method, page 5, line 97-104” |  |
| Quantitative variables | “Method, page 6/7, line 122-130” |  |
| Statistical methods | “Method, page 7/8, line 131-166” |  |
|  | “Method, page 7/8, line 131-166” |  |
|  | “Method, page 7/8, line 131-166” |  |
|  | “Not applicable” |  |
|  | “Not applicable” |  |
| **Results** | | |
| Participants | “Result, page 9, line 134” |  |
|  | “Not applicable” |  |
|  | (c) Consider use of a flow diagram |  |
| Descriptive data | “Result, page 9-11, line 174-187” |  |
|  | “no missed data” |  |
| Outcome data | “Result, page 9, line 134” |  |
| Main results | “Result, page 11-12, line 188-202” |  |
|  | (*b*) Report category boundaries when continuous variables were categorized |  |
|  | “Not applicable” |  |
| Other analyses | “Not applicable” |  |
| **Discussion** | | |
| Key results | “discussion, page 12, line 203-207” |  |
| Limitations | “discussion, page 15, line255-262 ” |  |
| Interpretation | “discussion, page 11-13, line 188-226” |  |
| Generalizability | “Conclusion page 16, line 264-272” |  |
| Other information |  |  |
| Funding | “Declaration, page 16, line 282-283” |  |
