# Supplementary material for: Individual and community-level factors influencing optimal breastfeeding: A multilevel analysis from a national survey study of Ethiopia
Source: PLoS One. 2021 Apr 29;16(4):e0241428. doi: 10.1371/journal.pone.0241428 (PMC8084135; doi:10.1371/journal.pone.0241428)
Supplement: S1 Appendix — (DOCX) [file pone.0241428.s004.docx]

**S1 Appendix.** Description of independent variables and their measurements used in the analysis.

| No | **Variables** | **Measurements** |
| --- | --- | --- |
|  | **Individual level variables** | |
| Child related variables | | |
| 1 | Child age | (1) < 2month, (2) 2-3 month, (3) 4-5 month |
| 2 | Sex of the child | (1)Male (2)female |
| 3 | Birth order | (1)first born, (2) second to fourth and (3) five or more |
| 4 | Size of child at birth | (1)large, (2) middle and (3) small |
| Mother’s characters | | |
| 1 | Mother’s age | (1) 15–19 years, (2) for 20–29, (3) for 30–39 and (4) for 40–49 years. |
| 2 | Educational status | (1) No education, (2) for primary education and (3) for secondary and above. |
| 3 | Occupation | 1. not employed and (1) employed |
| 4 | Religion | (1) Orthodox, (2)Muslim, (3) catholic and protestant (4) traditional and other |
| 5 | Marital status | (1)never married(2)married/living together and (3) divorced /separated/widowed |
| 6 | Mode of delivery | (0) non cesareanand (1) cesarean section |
| 7 | Place of delivery | (1) health facility (2) home and (3) other |
| 8 | Parity | (1)1-2 children, (2) 3-4 children and (3)>4 children |
| 10 | wealth index | (1) Poorest(2) poorer(3) middle income (4) richer (5) richest |
| 11 | Media exposure | (0) lacks access to all the three media (1) has access to all |
| 12 | - Ethnicity of mother | (1)Amhara, (2) Oromo, (3) Tigrie, (4)Affar (5)Somalia (6)other ethnic groups |
| 13 | Antenatal care | (1) No ANC, (2) 1 visit(3)2-3 visits (4)>=4 visits |
| Father’s characters | | |
| 1 | Father’s educational status | (1) No education (2) primary education (3) secondary and above. |
| 2 | Occupation | 1. not employed and (1) employed |
| **Community level Variables** | | |
| 1 | Residence | (1) Urban and (2) rural |
| 2 | Region | (1) Tigray, (2) Afar, (3) Amhara, (4) Oromo, (5) Somalia, (6) Benshangul, (7) SNNP, (8) Gambela, (9) Harare, (10) Addis Ababa and (11) Dire Dawa |
| **Community aggregate** | | The EDHS data did not collect data that can directly describe the characteristics of the clusters except place of residence and region. Therefore; the following community level variables was generated by aggregating individual level characters with our interest in cluster by using the proportion of given variable. Since their values are not normally distributed we use median value. |
| 3 | - Community ANC utilization | (0) Low =(<0.66) and (1) high=(>=0.66) |
| 4 | - Community media exposure | - (0) Low=(<0.24) and (1) high=(>=0.24) |
| 5 | - Community place of delivery | - (0) Low= (<0.333) and (1) high=(>=0.333) |
| 6 | - Community poverty | - (0) Low= (<0.555) and (1) high=(>=0.555) |
| 7 | - Community education | - (0) Low= (<0.333) and (1) high=(>=0.333) |
