# Supplementary material for: Individual and community-level factors influencing optimal breastfeeding: A multilevel analysis from a national survey study of Ethiopia
Source: PLoS One. 2021 Apr 29;16(4):e0241428. doi: 10.1371/journal.pone.0241428 (PMC8084135; doi:10.1371/journal.pone.0241428)
Supplement: S2 Appendix — (DOCX) [file pone.0241428.s005.docx]

**S2 Appendix. Model Specification.**

Four models containing variables of interest were fitted using xtmelogit command.

**Null Model (empty model):** Log**(**$\frac{\boldsymbol{y}_{\boldsymbol{ij}}}{\boldsymbol{1-}\boldsymbol{y}_{\boldsymbol{ij}}}$**)** = β_0j_+ u_0j_

Where, Log**(**$\frac{\boldsymbol{y}_{\boldsymbol{ij}}}{\boldsymbol{1-}\boldsymbol{y}_{\boldsymbol{ij}}}$**)** is the probability of optimal breastfeeding β_0j_is the regression intercept; ս_j_ is residuals at community level…………[1]..

This is the first step in the multilevel model analysis fitted without covariates to test random variability in the intercept and to estimate the intra-class correlation coefficient (ICC). The empty model enables the researcher to verify if the random effects at the community level are large enough to justify assessing random effects at the community level. When ICC is close to zero most of the variation is explained by lower-level which means there is no variation between-group effect and also if the ICC is close to one most of the variation is explained by a higher level that means there is no variation between lower-level effect [2]. However; the minimum value or cut point of ICC is 0.1 or 10% [3].

**Model I:** This model examines the effects of individual characteristics on optimal breastfeeding. These individual-level factors which were statistically significant at the bivariable analysis were included in this model. The equation is as follows: **Log (**$\frac{\boldsymbol{y}_{\boldsymbol{ij}}}{\boldsymbol{1-}\boldsymbol{y}_{\boldsymbol{ij}}}$**)** = β_0j_+ β_1j_X_1ij+…_β_nj_X_nij +_ u_nj +_ e_ij_

Where; **Log (**$\frac{\boldsymbol{y}_{\boldsymbol{ij}}}{\boldsymbol{1-}\boldsymbol{y}_{\boldsymbol{ij}}}$**)** = s the dependent variableβ_0j_=is intercept, ß_1j_= regression coefficient

X_1ij_= level-1 predictor _ij=_ is the random error --------------------------------------[1]

**Model II:** This model was used for community-level variables which were significant at the bivariable analysis. The equation looks as follows:

**Log (**$\frac{\boldsymbol{y}_{\boldsymbol{ij}}}{\boldsymbol{1-}\boldsymbol{y}_{\boldsymbol{ij}}}$**)** = β_0j_ + β_1j_Z_1j+…_β_nj_X_nj +_ u_nj +_ e_ij_

Where;**Log (**$\frac{\boldsymbol{y}_{\boldsymbol{ij}}}{\boldsymbol{1-}\boldsymbol{y}_{\boldsymbol{ij}}}$**) =** β_0j_ mean intercepts, β_1j_regression coefficients Z_nj_is community level explanatory variable and u_nj_= is the residual error at community level------------------------[1]

**Model III (Mixed model or final model):** this model includes both the individual and community level characteristics of optimal breastfeeding which were statistically significant and it allows to measure the effect of both individual and community characteristics simultaneously.

The final two models were expressed as **Log (**$\frac{\boldsymbol{y}_{\boldsymbol{ij}}}{\boldsymbol{1-}\boldsymbol{y}_{\boldsymbol{ij}}}$**) =** β_0j_ + β_1j_X_1j_ + β_1j_Z_1j +…+_ u_0j +_ e_ij_

β_0j_ is the intercept β_1 ,_ β_2,_β_n_ are the regression coefficients estimate the data X_1j,_ X_2j ,_X_nj_are covariates at individual level, Z_1j_covariate at community level-----[1]

**Parameter estimation methods**

Maximum likelihood estimators (MLE) maximize the probability of finding the sample data that actually found. The Maximum Likelihood (ML) was used to assess the goodness of diagnostic tests. This estimator includes both the regression coefficients and the variance components in the likelihood function [4]

In the multilevel models, the measures of association (fixed-effects) estimate the associations between the likelihood of children optimally breastfeed and various explanatory variables expressed as Adjusted Odds Ratio (AOR) with their 95% Confidence Intervals (CIs). The measures of variation (random-effects) were reported as Intra-class correlation coefficient (ICC) *ICC =* σ^2^u0 / (σ^2^u_0_ + σ e²) where σ e² = π^2^/3 where the variance was explained by the higher level. The change in the community level variance between the empty model (Model 1) and the consecutive model's V_e_ was expressed by Proportional Change in Community Variance (PCV) by using the formula PCV=(V_e_-V_mi_)/V_e_, The ICC and PCV were calculated at each model with reference to null model using the above formula.

**Model diagnostic**

The effect of multicollinearity between the predictor variables was checked using a variance inflation factor (VIF) at a cutoff point of 10. Predictors having a VIF value of less than 10 indicate an absence of Multicolliniarty [5]

**Model selection**

Akaike information criteria (AIC) were used to compare and check the goodness of fit of consecutive models. The AIC values for each model were compared and the model with the lowest value of AIC was considered to be a better explanatory model fitting the data very well [2]

**Model accuracy**

The receiver operating character (ROC) curve were used to show in a graphical way the connection /tradeoff between sensitivity and specificity for every possible cutoff for a test or combination of tests and the area beneath the curve is often used as a measure of the predictive power (usefulness of a test) which indicates the greater the predictive power, the more bowed the curve. A model with no predictive power has area 0.5; a perfect model has area 1. Lroc was used to examine the predictive ability of the model.

**References**

1. Guo G, Z.H., Multilevel modeling for binary data. Annual review of sociology, 2000. 26(1): p. 441-62.

2. Boedeker P, Hierarchical Linear Modeling with Maximum Likelihood, Restricted Maximum Likelihood, and Fully Bayesian Estimation. Practical Assessment, Research & Evaluation, 2017. 22(2): p. 2.

3. Shieh, G. Choosing the best index for the average score intraclass correlation coefficient. *Behav Res***48,**994–1003 (2016). https://doi.org/10.3758/s13428-015-0623-y

4. Steenbergen MR, Jones BS, Modeling multilevel data structures. American Journal of political Science, 2002: p. 218-47.

5. Craney TA, Surles JG. , Model-dependent variance inflation factor cutoff values. Quality Engineering, 2002. 14(3): p. 391-403.
